# Poster Presentations

**DOI:** 10.1080/135771402320883871

**Published:** 2002-09


### POSTER PRESENTATIONS

(In abstract number order)

### Dedifferentiated Chondrosarcoma: Updated Outcomes With Current Treatment Approaches As Compared To Those Prior To 1984

[Abstract ID: 10]

**Category:** Surgery
**Authors:** Ian Douglas Dickey[1], Peter S. Rose[1]
**Author Institutions:** [1]Mayo Clinic, Minnesota, United States
**Presenter:** Ian Douglas Dickey
dickey.ian@mayo.edu
**Correspondent:** Ian Douglas Dickey
dickey.ian@mayo.edu
Rochester Minnesota United States 55905
Ph: 507–538–2424
Fax: 57–284–5075

*Objectives:* Dedifferentiated chondrosarcoma presents a very difficult clinical problem. Long term survival is known to be poor, but a large clinical series has not been analyzed in the era of modern diagnostic and treatment modalities.
*Methods:* A retrospective chart review of all cases of patients presenting with dedifferentiated chondrosarcoma at our institution from 1984–2000 was performed. This was done as an extension to a study published in 1986 prior to the era of modern chemotherapy.
*Results:* There were 42 cases in 25 men and 17 women of average age 56 (range 24–83 years). MSTS grades at presentation were 5 IIA, 27 IIB, and 10 III. Three patients underwent biopsy only, 19 had limb sacrificing, and 20 had limb sparing procedures; surgical margins were intralesional in 3, marginal in 2, and wide in 20, and radical in 14. Twenty-seven patients received adjuvant therapy (22 chemotherapy only, 2 radiotherapy only, 3 combined therapy). Median survival was 8 months; 5-year survival was 7.1%. There was no statistical difference in survival between patients who did and did not receive chemotherapy, had wide versus radical resection, or had limb sparing versus sacrificing procedures. There were no statistically significant difference between patients treated prior to 1986 and those subsequently.
*Conclusions:* Despite advances in diagnostic modalities, surgical treatments, and adjuvant therapies, dedifferentiated chondrosarcoma continues to carry a poor prognosis. The use of current adjuvant chemotherapy and its inherent risks and benefits remains questionable in this population.

### Randomized Trial Of Amifostine With High Dose Alkylator Therapy In Ewing Sarcoma: A Pog/ccg Intergroup Study

[Abstract ID: 15]

**Category:** Pediatric Oncology
**Authors:** Mark L. Bernstein[1], Meenakshi Devidas[1]
**Author Institutions:** [1]Childrens Oncology Group, United States
**Presenter:** Mark L. Bernstein
bernstm@magellan.umontreal.ca
**Correspondent:** Mark L. Bernstein
bernstm@magellan.umontreal.ca
Montreal Quebec Canada H3T 1C5
Ph: 514–345–4969
Fax: 514–345–4792

*Objectives:* To determine if amifostine given twice with each dose of ifosfamide or cyclophosphamide could provide protection from the myelotoxic effects of high dosage alkylator therapy, and prevent delay in the administration of chemotherapy.
*Methods:* Patients less than 30 years of age with Ewing sarcoma metastatic at diagnosis and normal organ function were enrolled on P9457, a study of maximally intensified alkylator therapy administered without stem cell support. Cycles included alternating courses of ifosfamide, etoposide and vincristine, doxorubicin and cyclophosphamide. Amifostine was given to a randomized one-half of ____patients entered in the last half of the study. The dosage administered was 825 mg/m2 15 minutes prior to, and 3 hours after the beginning of each dose of ifosfamide or cyclophosphamide, with premedications and precautions as previously described. Days ANC <500/ul, platelets >50,000/ul and between cycles were analyzed at weeks 6, 12 and 18 of therapy.
*Results:* There was no significant difference seen in the arithmetic mean number of days with platelet count < 50,000/ul: 31 with amifostine, 30 without amifostine (Wilcoxon rank sum test, p-value: 0.38), the arithmetic mean number of days with absolute neutrophil count < 500/ul: with amifostine, 31 days, without amifostine, 30 days (WRS, p = 0.14), or the number of days until the next chemotherapy cycle: with amifostine: 30 days, without amifostine, 29 days (p= 0.45)
*Conclusions:* In the dose and schedule used, amifostine did not provide myeloprotection from the toxicity of high dose alkylator therapy, and did not shorten the interval until the administration of the next chemotherapy cycle.

### Cd44s Is A Prognostic Marker In Soft Tissue Sarcoma

[Abstract ID: 23]

**Category:** Surgery
**Authors:** Matthias Peiper[1], Takeo Sato[1], Antje Heinecke[1], Claus F. Eisenberger[1], Wolfram Trudo Knoefel[1], Jakob R. Izbicki[1]
**Author Institutions:** [1]Department of Surgery University of Hamburg, Germany **Presenter:** Matthias Peiper
peiper@uke.uni-hamburg.de
**Correspondent:** Matthias Peiper
peiper@uke.uni-hamburg.de
Hamburg Germany 20246
Ph: xx4949428032450
Fax: xx4940428033458

*Objectives:* The expression of CD44 has been identified as prognostic factor in several malignant diseases. However, only few data exist correlating CD44 expression in soft tissue sarcoma with subsequent tumor progression or recurrence. The purpose of this study was to investigate the clinical significance of CD44s in adult soft tissue sarcoma (STS).
*Methods:* Tumor specimens of 62 patients with STS were evaluated regarding CD44s expression using immunohistochemistry. The significance of the proposed prognostic indicators was evaluated in relation to survival and local recurrence.
*Results:* Of 62 analyzed specimens, 49 tumors were CD44s positive compared to 13 CD44s negative tumors. Kaplan-Meier survival analysis indicated significantly better survival among patients whose tumor was CD44s positive (P = 0.015). Variables predictive of longer survival included resection quality (R0, P < 0.01) and tumor size (T1, P = 0.02). CD44s expression correlates with prognosis of soft tissue sarcomas.
*Conclusions:* CD44s may play a pathogenetic role in tumor progression Determining the expression of CD44s in primary STS could be a valuable tool for selecting patients for further adjuvant treatment. Nevertheless, radical resection at initial surgery plays the pivotal role in soft tissue sarcoma treatment.

Immunohistochemical Basis For Combination Radioprotectant Treatment With Amifostine And Pentoxifylline For Growth Plate Protection During Irradiation
[Abstract ID: 28]

**Category:** Radiation Oncology
**Authors:** Timothy A. Damron[1], Sharad Mathur[1], Judy Strauss[1], Lee Reichel[1], Bryan Margulies[1], Yi Yang[1], Steve Landas[1], Cornelia Farnum[2], William Grant[1], Joseph A. Spadaro[1]
**Author Institutions:** [1]SUNY Upstate Medical University at Syracuse, New York, United States; [2]Cornell University College of Veterinary Medicine, New York, United States
**Presenter:** Timothy A. Damron
tdamron@twcny.rr.com
**Correspondent:** Timothy A. Damron
tdamron@twcny.rr.com
Syracuse New York United States 13202
Ph: 315–464–4472
Fax: 315–464–4664

*Objectives:* Irradiation of the growth plate during treatment of extremity sarcomas in pediatric patients often leads to undesirable late sequelae. Growth plate PTHrP has been reported to be down-regulated following irradiation. The purpose of this project was to test the hypotheses that PTHrP rebounds as part of the normal response following growth plate irradiation and that a proven radioprotectant may act to enhance this PTHrP response.
*Methods:* Sixty weanling 5 week Sprague-Dawley rats underwent right knee irradiation with single fraction 17.5 Gy while the left leg served as internal control. Twelve animals (half pretreated with amifostine) were euthanized at each of 0.5, 1, 2, 3, and 4 weeks. Immunohistochemical staining was performed and analyzed on tissue specimens for 3 animals per group per time for PTHrP, Bcl-2, Bax, caspase, TGF-beta and FGF-2.
*Results:* Irradiated tissue demonstrated reduced staining for PTHrP early, but by 2 weeks after irradiation there was a notable return in PTHrP expression to at least control levels. This post-irradiation PTHrP response was blunted rather than enhanced by amifostine. Amifostine showed its most dramatic effects in reducing Bax expression. Amifostine did not increase proliferative cytokines.
*Conclusions:* The observed PTHrP response corresponds to improving growth plate morphology and growth rate, suggesting a role for PTHrP in response to growth plate irradiation. Amifostine appears to decrease apoptosis mediators. This suggests a potential role for complementary radioprotectants that increase or maintain the post-irradiation PTHrP levels, such as pentoxifylline, as a means of maximizing growth plate recovery following irradiation.

Radiation Therapy In The Management Of Giant Cell Tumor Of Bone
[Abstract ID: 34]

**Category:** Radiation Oncology
**Authors:** James J Caudell[1], Matthew T Ballo[1], Gunar K Zagars[1], Valerae O Lewis[1], Rex A Marco[1], Kristin L Weber[1], Patrick P Lin[1], Robert S Benjamin[1], Adel K El-Naggar[1], Alan W Yasko[1]
**Author Institutions:** [1]UT MD Anderson Cancer Center, United States
**Presenter:** James J Caudell
jcaudell@mdanderson.org
**Correspondent:** Matthew T Ballo
mballo@mdanderson.org
Houston Texas United States 77030
Ph: 713–792–3400
Fax: 713–794–5573

*Objectives:* To evaluate the outcome for giant cell tumor of bone treated with radiation therapy, with or without surgical resection.
*Methods:* A retrospective review of 25 consecutive patients with pathologically confirmed giant cell tumor of bone receiving radiation therapy.
*Results:* The anatomic distribution of lesions was as follows: cervical spine, 3; temporal bone, 1; thoracic or lumbar spine, 9; sacrum, 8; iliac, 1; and one each in the humerus, radius, and 1st metacarpal bone. Tumor size ranged from 2–20 cm (median, 9.5 cm). Twelve patients were referred with recurrent disease having undergone one or more prior surgical resections or had been heavily pretreated using chemo-embolization with subsequent recurrence. Fourteen patients were treated for gross disease, the remaining eleven were treated after gross total surgical resection. The dose for radiation was 46 Gy (range 25–65 Gy). Median follow up was 8.8 years (0.67–34 years). The actuarial 5-year OS and DFS were 91% and 58%, respectively. The actuarial 5-year LC and DMFS rates were 62% and 81%, respectively. Univariate analysis suggested that treatment for recurrent disease correlated with an inferior DFS (33% vs. 83%, p=0.06), DMFS (64% vs. 100%, p=0.08), and LC rate (42% vs. 83%, p=0.08) at 5 years. Additionally, there was an inferior OS rate in those patients treated with radiation alone (80% vs. 100%, p=0.04).
*Conclusions:* Primary GCT of bone is a radiosensitive tumor. Radiation should be considered as an adjuvant to surgery or as an alternative in cases where excision would result in significant functional deficits.

Novel Radioprotectant Drugs Other Than Amifostine For Sparing Radiation-induced Damage To The Physis
[Abstract ID: 38]

**Category:** Radiation Oncology
**Authors:** Timothy A. Damron[1], Joseph A. Spadaro[1], Jason Horton[1], Bryan Margulies[1], Judy Strauss[1], Cornelia N. Farnum[2]
**Author Institutions:** [1]SUNY Upstate Medical University at Syracuse, New York, United States; [2]Cornell University College of Veterinary Medicine, New York, United States
**Presenter:** Timothy A. Damron
tdamron@twcny.rr.com
**Correspondent:** Timothy A. Damron
tdamron@twcny.rr.com
Syracuse New York United States 13202
Ph: 315–464–4472
Fax: 315–464–4664

*Objectives:* Amifostine, a selective free-radical scavenger, is the only previously documented drug to achieve radioprotection of the growth plate, but its effect is incomplete. The aim of this pilot is to determine if any of four novel radioprotectant drugs other than amifostine can individually preserve the integrity of, or minimize damage to, physeal longitudinal growth during single radiation dose exposure in an animal model.
*Methods:* Eighty-four weanling male Sprague-Dawley rats were randomized into 16 groups of 4–6 animals. All groups received single 17.5 Gy radiation exposure to the right knee with the left serving as control. Groups IA-C received pentoxifylline 50, 200, and 300 mg/kg. Groups IIA-B received selenium selenite 1.5 and 5 mg/kg. Groups IIIA-E received IL-1b 5, 15, 50, 125, and 500 mcg/kg. Groups IVA-E received misoprostol 0.1, 0.5, 1, 0, and 5 mg/kg. Group V received irradiation alone. At 6 weeks femoral and tibial lengths were measured and limb length percentage differences calculated between irradiated and unirradiated limbs. ANOVA was employed with alpha 0.05.
*Results:* The radiation dose of 17.5 Gy caused a mean femoral length discrepancy of 21.1%. Pentoxifylline at the lowest dose reduced mean femoral limb discrepancy to 15.3% (p=0.0001). Higher doses were less effective. IL-1beta at 15 mcg/kg reduced femoral mean length discrepancy to 16.7% (p<0.0001). Selenium and misoprostol demonstrated dose-dependent effects, the highest dose for each resulting in 17.2% discrepancy (p<0.03).

*Conclusions:* At specific doses, each of the four tested drugs, administered prior to a single irradiation dose, significantly reduced limb length discrepancy in our animal model. The magnitude of the reduction remains less than previously demonstrated for amifostine. Combination studies are underway.

Morphological And Biological Heterogeneity Of Three Tumorigenic Cell Lines Derived From A Single P53-/- Osteoblast-like Cell Line, Mmc2
[Abstract ID: 43]

**Category:** Medical Oncology
**Authors:** Koichi Nishijo[1], Tomitaka Nakayama[1], Hiroshi Murakami[1], Tomoki Aoyama[1], Takeshi Okamoto[1], Takashi Nakamura[1], Junya Toguchida[2]
**Author Institutions:** [1]Department of Orthopaedic Surgery Kyoto University, Kyoto Prefecture, Japan; [2]Institute for Frontier Medical Sciences Kyoto University, Kyoto Prefecture, Japan
**Presenter:** Koichi Nishijo
knsjo@frontier.kyoto-u.ac.jp
**Correspondent:** Junya Toguchida
togjun@frontier.kyoto-u.ac.jp
Kyoto Kyoto Prefectur Japan 606–850
Ph: +81–75–751–4134
Fax: +81–75–751–4144

*Objectives:* We describe three tumorigenic murine cell lines, which have a p53 deficient osteoblast-like cell as a common precursor.

*Methods:* MMC2, p53-/- osteoblast-like cell line, was innoculated subcutaneously into athymic mice. Cells isolated from tumors were cultured in vitro. These cells were assayed for growth properties in vitro and tumorigenicity in nude mice . Expression of osteoblast-related genes were examined by Northern blotting.

*Results:* Tumors developed in 3 out of 8 sites, and polyclonal cell lines were established from each tumor, and designated as MMOS1, MMOS2 and MMOS3. Expression patterns of the osteoblast-related genes were correlated well with the features of the original tumors, ranging from an osteoblastic osteosarcoma (MMOS2) to tumors with scarce or no osteoid formation (MMOS1 and MMOS3). Properties as malignant cells also varied among the three cell lines. MMOS1, which showed the slowest growth in a low-serum condition, developed markedly larger tumors in vivo than the other two cell lines. MMOS3 showed the fastest growth in a low-serum condition and produced the largest number of colonies in soft agar, but did not develop lung metastases, whereas MMOS1 and MMOS2 developed lung metastases with a frequency of 30% and 50%. Because these three cell lines have a common precursor, the heterogeneity among them is clearly attributed to genetic alterations that took place during transformation process, and therefore these cell lines will be suitable materials to isolate the responsible genes.

In Vitro Transformation Of The Rb(-/-) Murine Osteoblast
[Abstract ID: 44]

**Category:** Medical Oncology
**Authors:** Takeshi Okamoto[1], Hiroshi Yamamoto[1], Tomoki Aoyama[1], Koichi Nishijo[1], Takeharu Nakamata[1], Taisuke Hosaka[1], Tomitaka Nakayama[1], Takashi Nakamura[1], Junya Toguchida[2]
**Author Institutions:** [1]Department of Orthopaedic Surgery Kyoto University, Japan; [2]Instiitute for Frontier Medical Science Kyoto University, Japan
**Presenter:** Takeshi Okamoto
tokmt@frontier.kyoto-u.ac.jp
**Correspondent:** Junya Toguchida
togjun@frontier.kyoto-u.ac.jp
Kyoto city Japan 606–8507
Ph: +81–75–751–4142
Fax: +81–75–751–4144

*Objectives:* Objective: It is well known that the retinoblastoma (Rb) gene plays an important role in the development of osteosarcoma. Precise sequential events after the inactivation of the Rb gene, however, remains to be investigated. Here we have performed in vitro transformation experiments using Rb-/- murine osteoblasts as a starting material.

*Methods:* Rb-/- osteoblast-like cells were isolated from long bone of neonatal chimeric mice composed of wild-type and Rb-/- cells, and using the ALP activity as an osteoblast marker, one clonal cell line (¢ RbOB1) was established.

*Results:* ¢ RbOB1 expressed several marker genes as osteoblast (type I collagen, osteocalcin, and cbfa1/runx2), and showed vigorous growth in vitro, but neither anchorage-independent growth nor in vivo tumorigenecity was observed. To inactivate the p53 gene in the ¢ RbOB1, p53DD, the truncated dominant-negative form of human p53 gene, was introduced by the retrovirus vector, and a clonal cell line expressing p53DD was established (¢ RbOB1p53DD). Induction of p53 gene by actinomycin D was observed in the ¢ RbOB1, but not in the ¢ RbOB1p53DD, suggesting that the wild-type p53 was inhibited in ¢ RbOB1 p53DD. However, neither anchorage-independent growth nor in vivo tumorigenecity was observed in the {?} RbOB1p53DD. Finally, transfection of the oncogenic H-ras gene endowed ¢ RbOB1p53DD with the activity as completely transformed cells, but tumors developed in nude mice failed to form osteoid.

*Conclusions*: The inactivation of Rb and p53 in osteoblast wasn't enough for the development of osteosarcoma. Therefore, we still missed the last piece to develop osteosarcomas from osteoblasts.

External Beam Radiation Is An Effective Adjuvant Therapy For Clear Cell Sarcoma
[Abstract ID: 45]

**Category:** Radiation Oncology
**Authors:** James Hayden[2], David Zurakowski[3], Thomas Delaney[1], Francis Hornicek[1], Henry Mankin[1], Mark Gebhardt[1]
**Author Institutions:** [1]Massachusetts General Hospital, Massachusetts, United States; [2]Oregon Health and Sciences University, Oregon, United States; [3]The Childrens Hospital Boston, MA, United States
**Presenter:** James Hayden
haydenjam@hotmail.com
**Correspondent:** James Hayden
haydenjam@hotmail.com
Belmont MA United States 02478
Ph: 617–724–3700
Fax: 617–726–6823

*Objectives:* Clear cell sarcoma (CCS) is a rare soft tissue sarcoma. We sought to determine prognostic indicators and effective treatment methods.

*Methods:* We completed a retrospective review of the Massachusetts General Hospital and Children's Hospital, Boston, records identifying 21 patients. We completed a systematic review of the English literature identifying 283 patients with clinical data from 83 articles. The cumulative database was used to evaluate patient demographics, prognostic factors, and the effect of external beam radiation.

*Results:* Common locations were foot/ankle, 38.7 percent, knee, 12.4 percent, and wrist/hand, 12.4 percent. Common metastatic sites were lymph nodes and the lung. Overall 5 year survival was 48.6 percent. Metastasis at presentation was a prognostic indicator with 5 year survival of 13 percent vs 55 percent ($p < 0.0001$). Tumor size less than 5 cm was prognostic with 5 year survival rates of 30 percent vs 67 percent ($p < 0.0001$). 15 patients received external beam radiation for their primary tumor. All 15 had a surgical resection, were metastasis free at diagnosis, and did not receive chemotherapy. These patients received an average of 59.9 Gy. A control group of 100 patients with no radiation therapy but similar demographics (age, tumor size, primary tumor, and metastatic free at diagnosis) and treatment (surgical resection without chemotherapy) was identified. Fewer patients treated with radiation developed metastasis (13 vs 43 % $p < 0.05$) or died from disease (13 vs 43 % $p < 0.05$).
*Conclusions:* CCS tends to occur in young adults, in distal locations and metastasize to lymph nodes and lung. Tumor size equal or greater than 5 cm and metastasis at diagnosis are poor prognostic indicators. External beam radiation is an effective adjuvant for primary CCS therapy.

Expression Of The Chondromodulin-1 Gene In Osteosarcomas Suggests The Presence Of Osteo-chondral Bidirectional Precursor Cells
[Abstract ID: 51]

**Category:** Medical Oncology
**Authors:** Tomoki Aoyama[1], Takeshi Okamoto[1], Koichi Nishijo[1], Tatsuya Ishibe[1], Ko Yasura[1], Takeharu Nakamata[1], Taisuke Hosaka[1], Tomitaka Nakayama[1], Takashi Nakamura[1], Junya Toguchida[2]
**Author Institutions:** [1]Department of Orthopedic Surgery Kyoto University, Japan; [2]Insitute for Frontier Medical Science Kyoto University, Japan
**Presenter:** Tomoki Aoyama
blue@frontier.kyoto-u.ac.jp
**Correspondent:** Junya Toguchida
togjun@frontier.kyoto-u.ac.jp
Kyoto city Japan 606–8507
Ph: 81–75–751–4134
Fax: 81–75–751–4144

*Objectives:* Chondromodulin-1(ChM1) is a glycoprotein that inhibits angiogenesis and the expression is restricted to cartilage and eye. In cartilageous tumors, the expression of ChM1 was observed only in benign lesions, suggesting the role of loss of ChM expression during the malignant transformation (Hayami, et al. FEBS letter, 2001). No information, so far, has demonstrated concerning the expression of ChM1 in osteosarcomas (OS), and here we report that some OS express the ChM1, which may relate to the origin of tumor cells.
*Methods:* Using RT-PCR, the expression of mRNA of ChM1 in 26 cases of osteosarcoma tissues and 7 lines of osteosarcoma cell lines were analyzed.
*Results:* The mRNA expression of the ChM1 was observed in 10 out of 26 OS, most of which were subclassified as chondroblastic OS. Among seven OS cell lines, the expression of ChM1 was observed in two, one of which was established from a chondroblastic OS. Tumors positive for the ChM1 expressed the type II and IX collagen, and aggrecan genes, as well as the osteocalcin genes, suggesting the bidirectional differentiation potential of these tumor cells. Treatment of ChM1 negative OS cell lines with 5-azadeoxy-cytidine induced the expression of the ChM1 gene in two cell lines. Bisulfite sequencing demonstrated the methylated CpG residues in the critical promoter lesion in these cell lines, which were not methylated in human normal cartilage cells.
*Conclusions:* These data suggest that some OSs stem from the mesenchymal cells with bidirectional potential, and epigenetic mechanisms may take a part in the process of cell fate determination.

Malignant Fibrous Histocytoma Of The Extremities And Trunk – An Institutional Review
[Abstract ID: 52]

**Category:** Surgery
**Authors:** Alexandra Koenig[1], Matthias Peiper[1], Wolfram Trudo Knoefel[1], Jakob R. Izbicki[1]
**Author Institutions:** [1]Department of Surgery University Hospital Hamburg, Germany
**Presenter:** Alexandra Koenig
alexandra.koenig@gmx.de
**Correspondent:** Matthias Peiper
peiper@uke.uni-hamburg.de
Hamburg Germany 20246
Ph: 004940428032450
Fax: 004040428033458

*Objectives:* Malignant fibrous histiocytoma is the most common subtype of soft tissue sarcoma. Detailed understanding of this tumour type may lead to improved therapeutical strategies.
*Methods:* An institutional review was performed about all MFH patients operated on between 1988 and 1998.
*Results:* Eighty-six- patients with histologically confirmed MFH (G1: n=8, G2: n=23, G3: n=55) were analysed. Local recurrence was 36% after a median of 13 months. Distant metastases occurred in 29% of patients. After a mean follow-up of 5.5 years, 42 patients were alive without evidence of disease, median survival time was 68 months at a cumulative 5-year survival rate of 65%. Tumour size significantly influenced disease free survival (T2 vs. T1, $P < 0.01$, risk ratio [RR] 5.5), as did tumour depth (subfascial tumours, P = < 0.01, RR 3.3), and presence of lymph nodes ($P = 0.02$, RR 6.5). Positive microscopic margins and subfascial tumours were associated with an increased local recurrence rate (RR 5.7, $P < 0.0001$ and RR 3.5, $P = 0.02$, respectively). The only multivariate risk factors of distant metastases were tumour depth, in which patients with subfascial tumours fared worse (RR 4.0, $P < 0.01$) and tumour grade (RR 5.5, $P = 0.03$).
*Conclusions:* We conclude that aggressive but limb preserving resection of MFH should be performed at initial operation to minimize risk of local recurrence; a strict follow-up especially of subfascial tumours should be performed.

Proliferation And Apoptosis Are Independent Prognostic Parameters In Human Liposarcoma
[Abstract ID: 53]

**Category:** Surgery
**Authors:** Eike Gert Achilles[3], Sabine Lasch[1], David Zurakowski[3], J Schulz[1], Matthias peiper[1], W D Beecken[2], Xavier Rogiers[1]
**Author Institutions:** [1]Department of Surgery University Hospital Eppendorf, Germany; [2]Clinic for Urology University Hospital Frankfurt, Germany; [3]Department of Research Statistics Childrens Hospital Boston, United States
**Presenter:** Eike Gert Achilles
achilles@uke.uni-hamburg.de
**Correspondent:** Eike Gert Achilles
achilles@uke.uni-hamburg.de
Hamburg Germany 20246
Ph: 004940428032450
Fax: 004940428033458

*Objectives:* As an adjunct to conventional grading in human liposarcomas, the possible prognostic value of the rate of tumor cell apoptosis and proliferation was investigated.
*Methods:* 51 patients (female n=21, male n=30) with liposarcoma resected between 1988 and 2000 in our center were included in this study. Tumors were localised in the extremities (n=28), retroperitoneum (n=20) and trunk (n=3). Tumor margins were free (n=25) or showed residual microscopic (n=25) or macroscopic (n=1) disease. Multiple variables for each patient, including age, sex, tumor size, grading and numbers of apoptotic and proliferating cells were determined. Immunocytochemistry was performed for semiquantitative analysis of representative paraffin embedded tissue sections. Monoclonal antibodies targeted to proliferating cell nuclear antigen and apoptotic nuclei (Tunel-assay) were utilised. The results were correlated with the postoperative course of these patients

*Results:* The median survival time was 10.2 years. Multivariate analysis revealed a significant correlation between survival and grading, apoptosis and proliferation ($p < 0.05$ each).

*Conclusions:* These data indicate that in liposarcoma tumor cell apoptosis and proliferation are in addition to conventional grading of strong prognostic value in the assessment of disease- specific survival.

Reconstruction With Scapular Endoprosthesis Provides Superior Results After Total Scapular Resection: surgical Technique And Comparison To Patients Without Endoprosthetic Reconstruction [Abstract ID: 58]

**Category:** Surgery

**Authors:** Felasfa M Wodajo[1], Jacob Bickels[2], James C Wittig[3], Kristen Kellar-Graney[1], Yehuda Kollender[2], Isaac Meller[2], Martin M Malawer[1]
**Author Institutions:** [1]Washington Cancer Institute, DC, United States; [2]Tel Aviv Sourasky Medical Center, Israel; [3]NYU Medical Center Tisch Hospital, NY, United States
**Presenter:** Felasfa M Wodajo
felasfa@earthlink.net
**Correspondent:** Felasfa M Wodajo
felasfa@earthlink.net
Washington DC United States 20010
Ph: 202–877–7561
Fax: 202–877–8959

*Objectives:* Introduction
Suspension of the humeral head from the clavicle after total scapular resection is compared to endoprosthetic scapular reconstruction. The surgical technique of endoprosthetic reconstruction and functional results are described.
Patients: 23 patients with scapular tumors requiring total scapular resection were treated. Resection included 12 total scapulectomies and 11 en-bloc resections of the scapula and humeral head. Seven patients received endoprostheses. Four had prosthetic humeral heads suspended from the clavicle and 12 had suspension of the native humeral head. All patients were followed more than 2 years.

*Methods:* Endoprosthetic Surgical Technique Patient selection was crucial. All peri-scapular muscles were tumor-free. Resection was usually performed using a posterior approach. Most high-grade scapula tumors were resected with the proximal humerus. Smaller than a natural scapula, the prosthesis facilitated soft-tissue reconstruction and was multiply fenestrated for myodesis. It was placed on the serratus anterior and covered by the rhomboids, trapezius and latissimus. A curved humeral head prosthesis was cemented into the humerus and connected to the scapular prosthesis using Gore-tex™.

*Results:* There were no deep wound infections, failures, or secondary amputations. Elbow range-of-motion and hand dexterity were similar. Patients with scapular endoprosthesis had better active abduction (60 – 90 vs. 10 – 20). Patients with endoprosthetic reconstruction had a more natural contour. 6 patients with scapular prostheses (86%) and 10 patients with humeral suspensions (62%) had a good-to-excellent functional outcome.

*Conclusions:* Scapular endoprosthesis reconstruction is associated with better functional and cosmetic outcomes compared to simple humeral head suspension from the clavicle.

In Vitro Chemosensitivity Of Human Soft Tissue Sarcoma Tissue [Abstract ID: 63]

**Category:** Medical Oncology

**Authors:** Hideo Morioka[1]
**Author Institutions:** [1]Department of Orthopaedic Surgery School of Medicine Keio University, Tokyo, Japan; [2]Department of Surgery School of Medicine Keio University, Tokyo, Japan
**Presenter:** Hideo Morioka
morioka@sc.itc.keio.ac.jp
**Correspondent:** Hideo Morioka
morioka@sc.itc.keio.ac.jp
Tokyo Tokyo Japan 160–8582
Ph: +81–3–5363–3812
Fax: +81–3–3353–6597

*Objectives:* The Histoculture Drug Response Assay (HDRA) is an in vitro chemosensitivity test that has a high correlation with clinical response, the usefulness of which has been reported in various kinds of solid tumors. In this study, in order to investigate the variation in chemosensitivity in STS, fresh biopsy or surgical samples of STS were tested using the HDRA methods.

*Methods:* Eighty samples of fresh human STS were obtained during either the biopsy or surgical removal at Keio University Hospital in Japan between 1997 and 2001. As anti-tumor drugs, cisplatin (CDDP), doxorubicin (ADM), pirarubicin (THP), 4-hydroxy-ifosfamide (4-H-IFO) and etoposide (VP-16) were used. HDRA was performed according to the method previously reported.

*Results:* Drug sensitivity testing by HDRA showed that two drugs, ADM and THP, had a significantly higher inhibitory rate than CDDP, IFOS, or VP-16 in the eighty soft tissue sarcomas tested. Depending on the morphological type, spindle cell sarcomas were sensitive to THP, which showed significantly higher inhibition rates than CDDP, IFOS, or VP-16. Small round cell sarcomas were relatively sensitive to all of the drugs tested. However the drug sensitivity of pleomorphic cell sarcoma was low except for ADM and THP, while its sensitivity to THP was higher than about 70%.

*Conclusions:* Depending on the morphological type, STS showed various chemosensitivity in HDRA. However, there are numerous other soft tissue sarcomas that do not belong to these categories; drug sensitivity testing in each of them and the devising of individualized treatment strategies seems necessary to improve the therapeutic outcome.

The Importance Of Tumour Volume To Distal Femoral Volume Ratio In The Presentation Of Giant Cell Tumours Of The Distal Femur
[Abstract ID: 64]

**Category:** Surgery

**Authors:** Lee Jeys[1], Raj Suneja[1], Simon Carter[1], Robert Grimer[1]
**Author Institutions:** [1]Royal Orthopaedic Hospital Oncology Service, Birmingham, United Kingdom **Presenter:** Lee Jeys
lee.jeys@btclick.com
**Correspondent:** Lee Jeys

*Objectives:* To identify the incidence of a cortical breech on the initial presentation X-rays of patients with distal femoral GCTs, and whether this lead to a higher rate of local recurrence of tumour and increase in severity of surgery.

*Methods:* A prospective database is kept of all patients seen in the unit, it contains data on over 10, 000 patients seen over 34 years. Using the database initial presentation X-rays on 54 patients with distal femoral GCTs were reviewed. The size of the tumour was estimated using a facility of the database, by measuring the largest dimensions of the tumour (depth, breadth & height). The volume of the distal femur was estimated using the same X-ray and computer programme. The X-rays were then carefully studied for evidence of a cortical breach on antero-posterior (AP) and lateral views. The records were also checked for evidence of subsequent locally recurrent disease and subsequent surgery.
*Results:* X-rays were reviewed on 54 patients (29 male, 25 female), range of 18–72 years. All patients had a biopsy proven GCT of the distal femur, X-rays (prior to biopsy) were reviewed. 34 (63%) patients with a cortical breech on X-ray. The mean tumour volume : distal femoral volumes (TV:DFV) was statistically greater between those patients with a cortical breach and those without, using ANOVA (p<0.0001). There were 13 patients with local recurrent disease but no statistical difference in subsequent local recurrence rates between the two patient groups. There was also no statistical differences between the number of operations for those who presented with a cortical breach or without. There was no evidence that more radical surgery was required if a patient presented with a cortical breach.
*Conclusions:* The risk of cortical breech in patients with GCTs of the distal femur is dependant upon the tumour volume to distal femur volume ratio. If the ratio is above 54% then present with a cortical breech on X-ray is likely, (95% confidence interval), conversely if the ratio is less than 44 % then a cortical breach is unlikely. There is no evidence those patients with a cortical breach have a higher rate of local recurrence, an increased number of operations or more radical surgery.

Low Local Recurrence Rate Of Large, Deep-seated, Soft-tissue Sarcomas With Resection After Induction (neoadjuvant) Chemotherapy: histological Analysis Of Capsule Formation And The Role Of adjuvant Radiotherapy
[Abstract ID: 65]

**Category:** Medical Oncology
**Authors:** Felasfa M Wodajo[1], Kristen L Kellar-Graney[1], James C Wittig[2], Kari L Mansour[1], Dhruv Kumar[1], Dennis A Priebat[1], Robert M Henshaw[1], Martin M Malawer[1]
**Author Institutions:** [1]Washington Cancer Institute, Washington, DC, United States; [2]NYU Medical Center Tisch Hospital, New York, United States
**Presenter:** Felasfa M Wodajo
felasfa@earthlink.net
**Correspondent:** Felasfa M Wodajo
felasfa@earthlink.net
Washington DC United States 20010
Ph: 202–877–3970
Fax: 202–877–8959

*Objectives:* Surgical resection with “wide” margins or a rim of normal tissue is the mainstay of local control for high-grade extremity soft-tissue sarcomas.
*Methods:* From 1988–2002, 56 patients completed neoadjuvant/adjuvant a chemotherapy regimen of continuous intravenous doxorubicin, and intra-arterial cisplatin and, after 1996, intravenous ifosfamide. No patient received preoperative radiotherapy. LR analysis was performed on 42 patients and 23 patients were used for analysis of “capsule” formation and development of a classification system. Type III indicated a well-developed fibrous rim with inner/outer reactive zones, Type II implied interrupted fibrous zone and type I described no outer reactive zone and ill-defined transition zone.
*Results:* Median follow-up was 55 months. LR was 12% (5/42). Local control analysis = 89.6% (95% CI: 74.7% – 96.2%: 22 months) 89.6% (95% CI:67.1.8% –.97.3%: 55 months) DFS was 73.7% (95% CI: 60% – 83.9%) and 65.5% (95% CI: 47.3% – 80%).OS was 90.4% (95% CI:79% – 96%) and 84.8% (95% CI: 67.5% – 93.7%). Disease-specific OS was 90.4% (95% CI: 78.9% – 95.9%) and 86.8% (95% CI: 69.6% – 94.9%). Average necrosis was 74%. Only 37.5% (21/56) received adjuvant radiation. Average tumor size was 11.8 cm. 7/56 (12.5 %) reported minor wound complications. 25 (60%) had reports specifying margin depth in centimeters. 21 (84%) reported </= margins specified had (60%) patients LR Most “tumor-free”. were remaining The focally-present. tumor (4%) 25 1 and margin to the from 0.5-cm/= 0.5 cm. Type III capsule formation correlated to 94.3% average necrosis (range 85%-99%), type II to 77% (range 50%-98%), type I to 34% (range 10–50%).
*Conclusions:* Surgical margins are reported as being predictive of local recurrence after soft-tissue sarcoma resection. Presence of close or focally positive margins in this series did not correlate with local recurrence. A well-formed fibrous “capsule” tumor periphery and good chemotherapeutic response may contribute to improved local control.

Gastrointestinal Stromal Tumour: The Patient Experience
[Abstract ID: 70]

**Category:** Medical Oncology
**Authors:** Anne McTiernan[1], Robyn Reagon[1], Jeremy Whelan[1]
**Author Institutions:** [1]The London Bone and Soft Tissue Tumour Service, London, United Kingdom **Presenter:** Anne McTiernan
anne.mctiernan@uclh.org
**Correspondent:** Anne McTiernan
anne.mctiernan@uclh.org
London United Kingdom W1T 3AA
Ph: +44 20 7387 9300 x3133
Fax: +44 20 7380 9321

*Objectives:* The management of patients with GIST has been fundamentally altered by the introduction of Imatinib. Results in advanced disease have shown dramatic results for a previously untreatable condition with little major toxicity. The experience of patients may be complex and varied, including emotions stemming from unexpected renewed health and uncertainty associated with long term treatment with a new drug. The observations of a clinical trial nurse have identified particular patterns: a first generation of patients diagnosed before Imatinib but who subsequently benefited from it and a second generation offered treatment from the time of diagnosis or relapse.
*Methods:* Illustrative case histories include 2 patients diagnosed before the introduction of Imatinib and had faced a terminal illness and 2 patients referred at diagnosis or at relapse specifically for Imatinib.
*Results:* Responses were partial remission in 2 and stable disease in 2. Both patients in the first group had a good symptomatic response with only grade 1–2 toxicities. One stated that any side effect would be acceptable to achieve benefit. The other resumed full time work but remains anxious about how long the response may last. In the second group, one patient had multiple grade 1–2 toxicities similar to those of her disease causing constant fears of tumour re-growth. Another had no side effects but anxieties from living with a cancer still in situ. All exhibited anxiety despite the success of treatment.
*Conclusions:* Health professionals caring for patents with GIST should recognise and acknowledge the additional issues faced by patients which accompany response to Imatinib.

Gemcitabine And Docetaxel In Sarcoma
[Abstract ID: 80]

**Category:** Medical Oncology
**Authors:** Kirsten M. Leu[1], Mark Zalupski[1], Vernon Sondak[1], Krisinda Snyder[1], Laurence H. Baker[1]

**Author Institutions:** [1]University of Michigan Comprehensive Cancer Center, MI, United States **Presenter:** Kirsten M. Leu
kmleu@umich.edu
**Correspondent:** Kirsten M. Leu
kmleu@umich.edu
Ann Arbor MI United States 48109–0948
Ph: 734/936–3983
Fax: 734/93607376

*Objectives:* A recent clinical trial of the combination of gemcitabine and docetaxel reported favorable results in patients with unresectable, predominantly uterine leiomyosarcoma (LMS). The objective of this report is to describe additional experience with this combination in a variety of histologic subtypes of sarcoma.
*Methods:* A retrospective chart review of 24 consecutively treated patients was performed.
*Results:* Twenty four patients with a median age of 52.5 years (range 22–70) were treated with gemcitabine 675 mg/m2 on days 1 and 8 and docetaxel 100 mg/m2 on day 8 of a 21-day cycle. Nineteen patients had previously received adriamycin, ifosfamide, or both. Eighteen patients had metastatic disease, 4 had locally recurrent disease, and 2 patients presenting with their original diagnosis were unable to receive adriamycin and/or ifosfamide for medical reasons. Patients received a median of 6 cycles of chemotherapy (range, 2–8 cycles); 5 continue on treatment at the time of this report. Responses occurred in 6/10 LMS from various primary sites, 2/2 angiosarcomas, 1/2 osteosarcomas, 1/2 malignant peripheral nerve sheath tumors, 0/2 synovial sarcomas, 1/2 malignant fibrous histiocytomas, 1/1 Ewing's sarcoma, 1/1 high grade sarcoma, not otherwise specified, 0/1 chondrosarcoma, and 0/1 liposarcoma. We observed 5 complete responses and 8 partial responses for an overall response rate of 54%. We are currently confirming these responses using the RECIST criteria.
*Conclusions:* The combination of gemcitabine with docetaxel is a potentially useful therapy for a variety of sarcomas. We look forward to participating in a multicenter clinical trial of this promising combination.

